# Acral melanoma detection using a convolutional neural network for dermoscopy images

**DOI:** 10.1371/journal.pone.0193321

**Published:** 2018-03-07

**Authors:** Chanki Yu, Sejung Yang, Wonoh Kim, Jinwoong Jung, Kee-Yang Chung, Sang Wook Lee, Byungho Oh

**Affiliations:** 1 Department of Media Technology, Graduate School of Media, Sogang University, Seoul, Republic of Korea; 2 Medical Physics Division, Department of Radiation Oncology, Stanford University School of Medicine, Palo Alto, United States of America; 3 Department of Electronics Engineering, Ewha Womans University, Seoul, Republic of Korea; 4 Department of Dermatology, Keimyung University, College of Medicine, Daegu, Republic of Korea; 5 Department of Dermatology and Cutaneous Biology Research Institute, Yonsei University College of Medicine, Seoul, Republic of Korea; University of Queensland Diamantina Institute, AUSTRALIA

## Abstract

**Background/Purpose:**

Acral melanoma is the most common type of melanoma in Asians, and usually results in a poor prognosis due to late diagnosis. We applied a convolutional neural network to dermoscopy images of acral melanoma and benign nevi on the hands and feet and evaluated its usefulness for the early diagnosis of these conditions.

**Methods:**

A total of 724 dermoscopy images comprising acral melanoma (350 images from 81 patients) and benign nevi (374 images from 194 patients), and confirmed by histopathological examination, were analyzed in this study. To perform the 2-fold cross validation, we split them into two mutually exclusive subsets: half of the total image dataset was selected for training and the rest for testing, and we calculated the accuracy of diagnosis comparing it with the dermatologist’s and non-expert’s evaluation.

**Results:**

The accuracy (percentage of true positive and true negative from all images) of the convolutional neural network was 83.51% and 80.23%, which was higher than the non-expert’s evaluation (67.84%, 62.71%) and close to that of the expert (81.08%, 81.64%). Moreover, the convolutional neural network showed area-under-the-curve values like 0.8, 0.84 and Youden’s index like 0.6795, 0.6073, which were similar score with the expert.

**Conclusion:**

Although further data analysis is necessary to improve their accuracy, convolutional neural networks would be helpful to detect acral melanoma from dermoscopy images of the hands and feet.

## Introduction

In Asians, melanoma is rare, compared to its prevalence in Caucasians, and usually occurs in acral areas such as the hands and feet. It can be misrecognized as benign nevi (BN), is occasionally hidden by calluses, and eventually results in late diagnosis at an advanced stage, with a poor prognosis[1–3]. Since effective anti-cancer agents for treating melanoma have not yet been developed, early detection and wide excision of the skin lesion is more crucial to the cure for melanoma.

Recently, to aid the early diagnosis of melanoma and the reduction of unnecessary skin biopsy, dermoscopy has been widely used[4, 5]. Moreover, because it is difficult for non-experts to use[6], artificial intelligence and deep-learning models have been applied to help physicians who are untrained to handle a digital dermoscope[7]; its use is expected to increase in the field of teledermatology.

A convolutional neural network (CNN) is one of the representative models among the various deep-learning models. It has already shown potential for general and highly variable tasks across many fine-grained object categories[8–12] and has been shown to exceed human performance in object recognition[9]. Recently, it was applied to detect skin cancers in images, including from dermoscopy, and successfully demonstrated artificial intelligence capable of classifying skin cancer with a competence level comparable to that of dermatologists[13]. For the success of CNN models, a large amount of training data labeled with class types to produce a rich feature hierarchy is necessary, and therefore, its usefulness in the diagnosis of rare diseases with insufficient data has not been fully established.

In this study, we applied an end-to-end CNN framework to detect a rare disease in Asians, acral melanoma (AM), from the dermoscopy images of pigmentation on the hands and feet. To overcome the insufficiency of the datasets, we adopted a transfer learning technique to leverage learned features from a CNN model pre-trained on a large-scale natural image dataset[14]. Moreover, we also applied a half-training and half-trial method to validate its clinical usefulness for the early diagnosis of patients compared with the dermatologist’s and non-expert’s evaluation.

## Methods

### 1. Dermoscopy images

**Fig 1:**
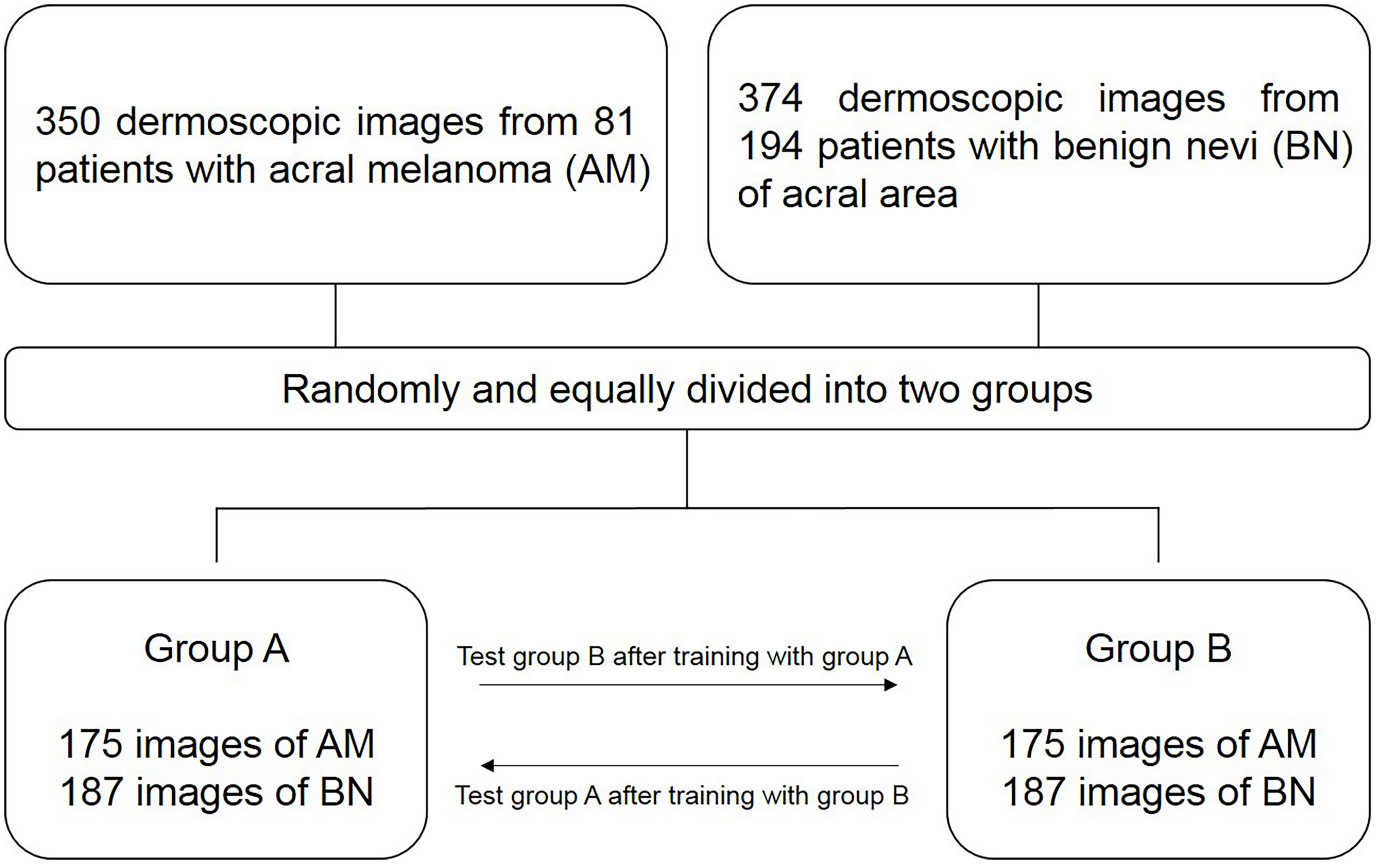
Flowchart of this study.

A total of 724 dermoscopy images were collected from January 2013 to March 2014 at the Severance Hospital in the Yonsei University Health System, Seoul, Korea, and from March 2015 to April 2016 at the Dongsan Hospital in the Keimyung University Health System, Daegu, Korea. Among them, 350 dermoscopy images were from 81 patients with AM and 374 images were from 194 patients with BN of the acral area (Fig 1). A total of 632 dermoscopy images were captured by the DermLite Cam (3Gen Inc., USA), and 92 images were captured by the Dermlite hybrid II (3 Gen Inc., USA), connected to a digital camera (Nikon Coolpix P6000, Japan). All diagnoses were histopathologically confirmed and multiple images were captured in cases of large lesions. We provide a STROBE checklist for the study of diagnostic efficacy as supporting information (S1 Table). Dermoscopy images of BN were divided into nine types, and AM images into three types according to the reference[15], by two dermatologists. This study protocol was approved by the Institutional Review Board of Yonsei University, Severance Hospital and Keimyung University, Dongsan Hospital and was conducted according to the Declaration of Helsinki Principles. Patient records/information was anonymized and de-identified prior to analysis.

### 2. Convolutional neural network to detect melanoma

We have described the CNN architecture we adopted in Section 2. 1 and presented the training and inference methods for detecting melanoma in Section 2. 2.

#### 2.1 Convolutional neural network

**Fig 2:**
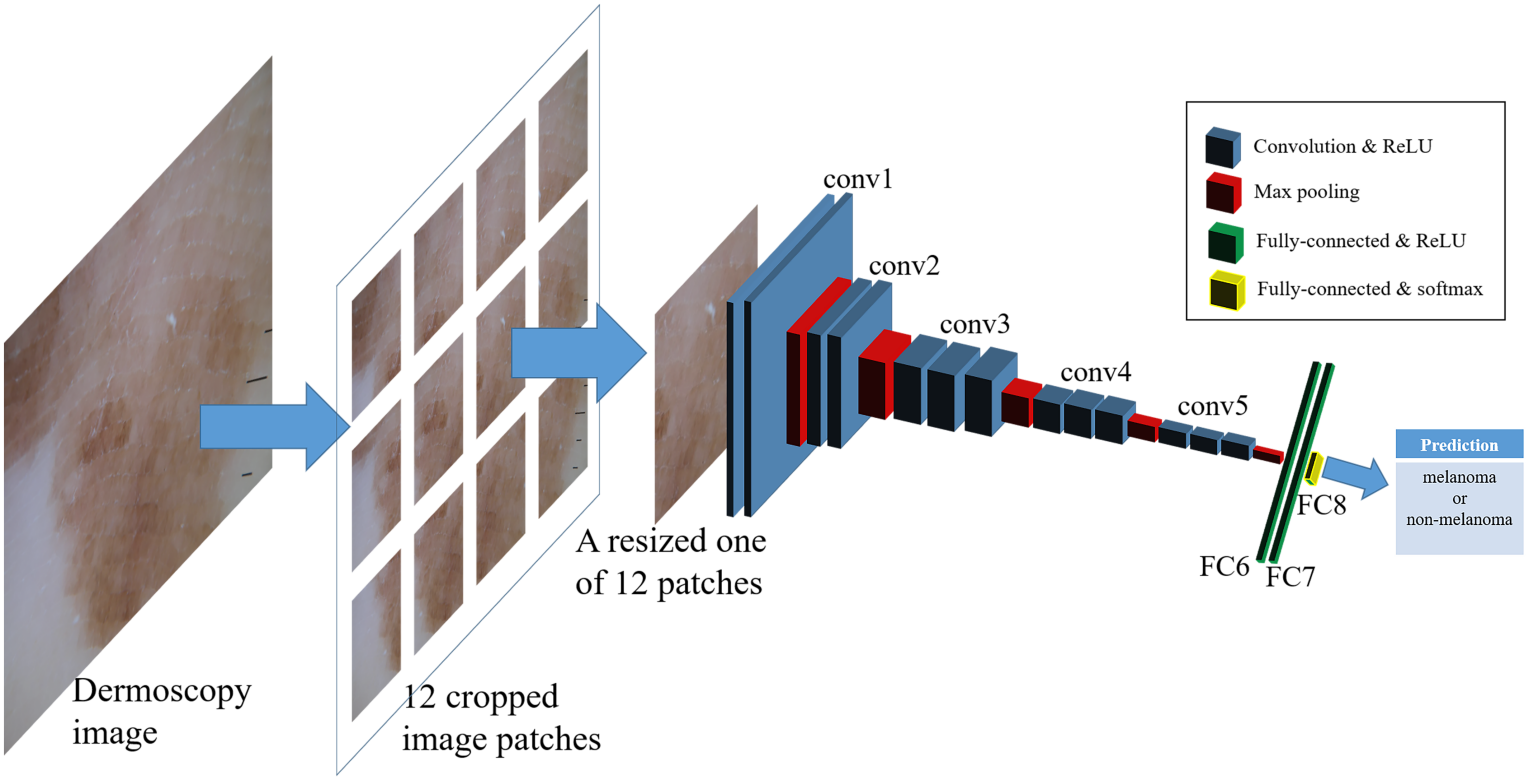
Schematic overview of our CNN architecture: The number of output classes was set to 2 (melanoma and non-melanoma classes) for the dermoscopic images.

CNNs are composed of several convolutional layers, each involving linear and nonlinear operators, as well as fully connected layers. The architecture for the state-of-the-art CNN has many parameters; for example, the VGG-16 Model has 138 million parameters, where the parameters are learned from the ImageNet dataset containing 1.2 million general object images of 1,000 different object categories for training[16]. Deep neural networks are difficult to train using small datasets (i.e., a few hundred images). To circumvent this problem, we used the fine-tuning technique, which is one of the regularization techniques. We fine-tuned a modified VGG model with 16 layers (13 convolutional and three fully connected layers), which uses the convolution filters of the same size (i.e., 3 × 3) for all convolution layers, as seen in Table 1. Our network configuration is depicted in Fig 2 and Table 1. Each layer and feature map in the CNN is represented by a three-dimensional array of size <*h* × *w* × *d* >, where *h* and *w* are spatial dimensions, and *d* is the number of channels or feature dimensions. The first layer is the input image patch with pixel size *h* × *w*, and *d* is the color channel (Fig 2 and Table 1; “conv” represents a convolutional layer and “FC” represents a fully connected layer).

**Table 1:** Our CNN configuration with 16 weight layers.

name	layer type	filter kernel size	feature map size
	Input		224×224×3
conv1	conv	3×3×64	224×224×64
	conv	3×3×64	224×224×64
	max-pooling		
conv2	conv	3×3×128	112×112×128
	conv	3×3×128	112×112×128
	max-pooling		
conv3	conv	3×3×256	56×56×256
	conv	3×3×256	56×56×256
	conv	3×3×256	56×56×256
	max-pooling		
conv4	conv	3×3×512	28×28×512
	conv	3×3×512	28×28×512
	conv	3×3×512	28×28×512
	max-pooling		
conv5	conv	3×3×512	14×14×512
	conv	3×3×512	14×14×512
	conv	3×3×512	14×14×512
	max-pooling		
FC6	fully-connected	1×1×4096	4096
FC7	fully-connected	1×1×4096	4096
FC8	fully-connected	1×1×2	2
	soft-max activation function		

The input with a fixed-size, 224 × 224, was passed through a stack of convolutional layers, where each followed a rectified linear unit (ReLU) activation function, and max-pooling was performed over a 2 × 2 pixel window with a stride of 2. A series of convolutional layers (conv1, conv2, conv3, conv4, and conv5) were followed by three fully connected layers: the first 2 fully connected layers (FC6 and FC7) had 4,096 channels each, where each followed a ReLU activation function, while the last fully connected layer (FC8) had 2 channels since our problem was a two-way classification problem (melanoma and non-melanoma class). It should be noted that the number of channels of the last fully connected layer was the same as the number of classes. Hence, we replaced the original fully connected layer (FC8: FC with 1000 channels) with a fully connected layer with two channels. The last layer had the soft-max activation function and predicted whether the input patch was a melanoma or non-melanoma lesion.

Moreover, the VGG-16 model pre-trained on the ImageNet database are used to perform transfer learning, and the weights of the last convolutional layers (the last two layers of conv5) and three fully convolutional layers (FC6, FC7, and FC8) are initialized using Xavier weight initialization[17]. In order to perform fine-tuning, we froze the weights of conv1, conv2, conv3, conv4, and the first layer of conv5 on pre-trained ImageNet, and trained the initialized weights on our dermoscopy image dataset. The above procedure is performed to prevent the large gradient caused by randomly initialized weights from ruining the pre-trained weights. After several training epochs, we trained the all weights of our network without freezing any layer.

#### 2.2 Training and inference

**Fig 3:**
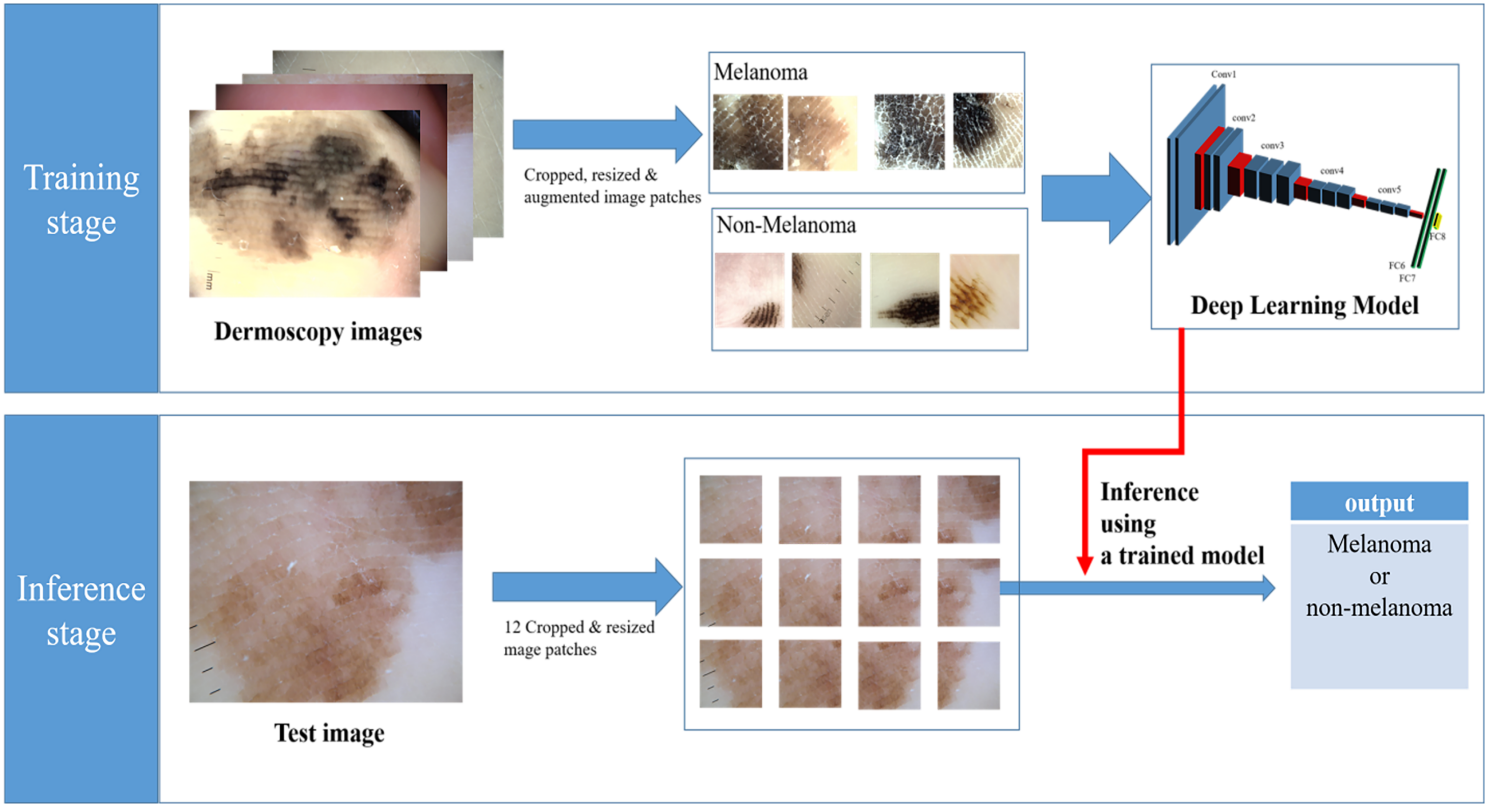
The framework of the melanoma classification showing training (upper) and inference (lower) stages.

Our dataset consisted of 724 images and associated labels, which were split into two mutually exclusive subsets (group A and B); half of the total image dataset was selected for training and the rest for testing. The scale and location of a skin lesion in a captured image were changed according to the capture conditions. To resolve this issue, we adopted a sliding window strategy and used the cropped patches instead of the full image at the training and inference time. At the inference time, we extracted about 12 image patches from each test image on a regularly spaced grid with a partial overlap between neighboring patches and then each patch was rescaled to the size of 224 × 224 pixels, as seen in Fig 3. In addition, to increase the robustness of the variation of geometric transformation in our CNN model, the training dataset was artificially augmented at training. Additional augmented data were formed by rotating and flipping images from the original training set. We generated 216 image patches from a single image using rotations by 0°, 45°, 90°, and 135°, as well as left-right and top-bottom reflections. In addition, the patches that did not contain any melanoma lesions among the melanoma training images were manually removed and the patches that did not contain any skin lesions among the non-melanoma training images were assigned to the non-melanoma class at training time.

We randomly selected 30% of the training dataset as a validation set and the rest as a training set at the onset of training. The validation data were used to prevent the overfitting of the training data and to provide guidance on when to stop training the network. The training of our CNN was stopped when the validation error on the validated dataset stopped decreasing. We trained the network using an adaptive stochastic sub-gradient method where the batch size is set to 50, and the momentum parameter, learning rate, and weight decay are set to 0.9, 0.0001, and 0.0005, respectively.

**Fig 4:**
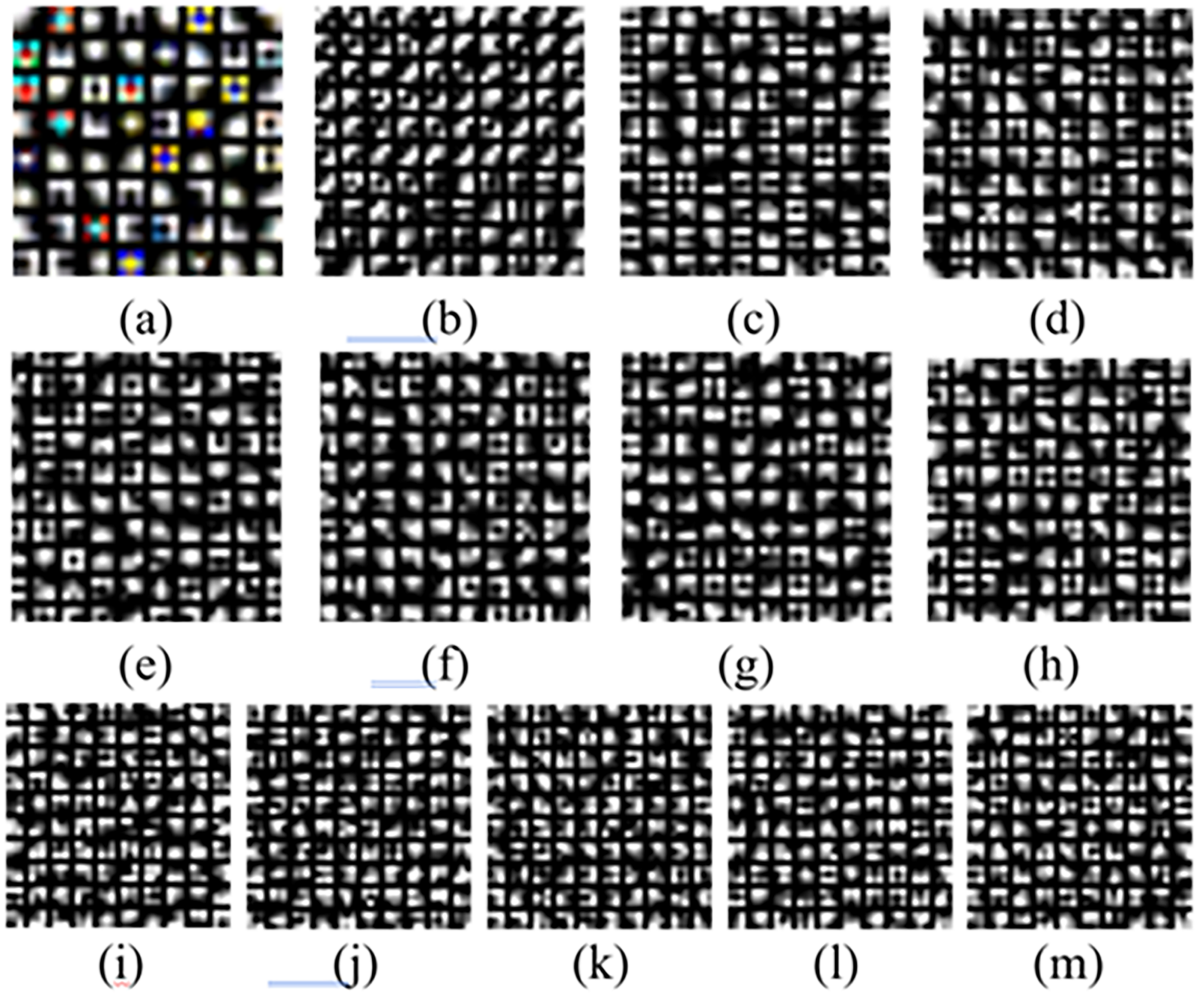
Visualization of the learned filters: (a) 64 learned filters at the first layer, (b-m) 100 filters among the learned filters from the 2nd to 13th layers, respectively.

Some of the filters learned from our melanoma dataset may be seen in Fig 4. Fig 4(a) shows 64 learned filters at the 1^st^ convolutional layer, where each represents a learned filter with a 3 × 3 kernel size. The input of the first layer is an RGB image with 224x224 pixel size, and it is convolved with 64 learned filters with 3x3 kernel size as shown in Fig 3(a) and 64 feature maps with 224x224 size are generated. In addition, the output feature maps are used as the input of the next layer. Fig 4(b)–4(m) shows 100 filters among the learned filters from the 2^nd^ to the 13^th^ convolution layer, respectively, where each represents a learned filter with a 3 × 3 kernel size. At the time of inference, we interpreted 12 image patches per test image, and when one or more images were predicted as containing melanoma, the corresponding test image was interpreted as containing melanoma. Each input of the network was an RGB image subtracted from the average image and calculated over the entire training image dataset. We implemented our method using MatConvNet, a Matlab-based CNN framework for computer vision applications[18]. Moreover, we fine-tuned a VGG model with 16 layers downloaded from http://www.vlfeat.org/matconvnet/pretrained).

### 3. Comparison of diagnostic rate

To assess the clinical usefulness of the CNN, we compared its diagnostic rate with those of two dermatologists who had five or more years of clinical experience in dermoscopy (expert group) and two non-trained general physicians (non-expert group). All images on the computer screen were evaluated simultaneously. If there was a dissensus between two physicians, they reached a conclusion under the agreement. Since 724 images were randomly and equally divided into two groups for the training of the CNN, we evaluated them separately as group A and B.

Based on the histopathologic results, we calculated the sensitivity, specificity, positive predictive value (PPV), negative predictive value (NPV), accuracy, Youden’s index, and area under the curve (AUC) as follows.

$$\text{Youden}’\text{s}\;\text{index}=\text{Sensitivity}\left(\frac{true\;positive}{true\;positive+false\;negative}\right)+\text{Specificity}\left(\frac{true\;negative}{true\;negative+false\;positive}\right)–1$$ (1)

$$\text{Accuracy}=\frac{true\;positive+true\;negative}{true\;positive+true\;negative+false\;positive+false\;negative}\times 100\left(\text{\%}\right)$$ (2)

The agreement between the pathologic result and each rater’s diagnosis was measured using the calculation of Cohen’s kappa coefficient. All statistical analyses were performed with MedCalc software version 17.9.

$$\text{Cohen}’\text{s}\;\text{Kappa}=\frac{Po-Pe}{1-Pe}$$ (3)

(Po = Accuracy, Pe = hypothetical probability of a chance agreement)

## Results

Among 724 dermoscopy images, 71 images were from the hands and fingers, and the others were from the feet and toes. A total of 350 AM images included homogenous diffuse irregular pigmented, parallel ridge, and multicomponent patterns, while 374 BN images included parallel furrow, fibrillar, lattice-like, reticular, globular, and homogenous patterns (S1 Table).

**Fig 5:**
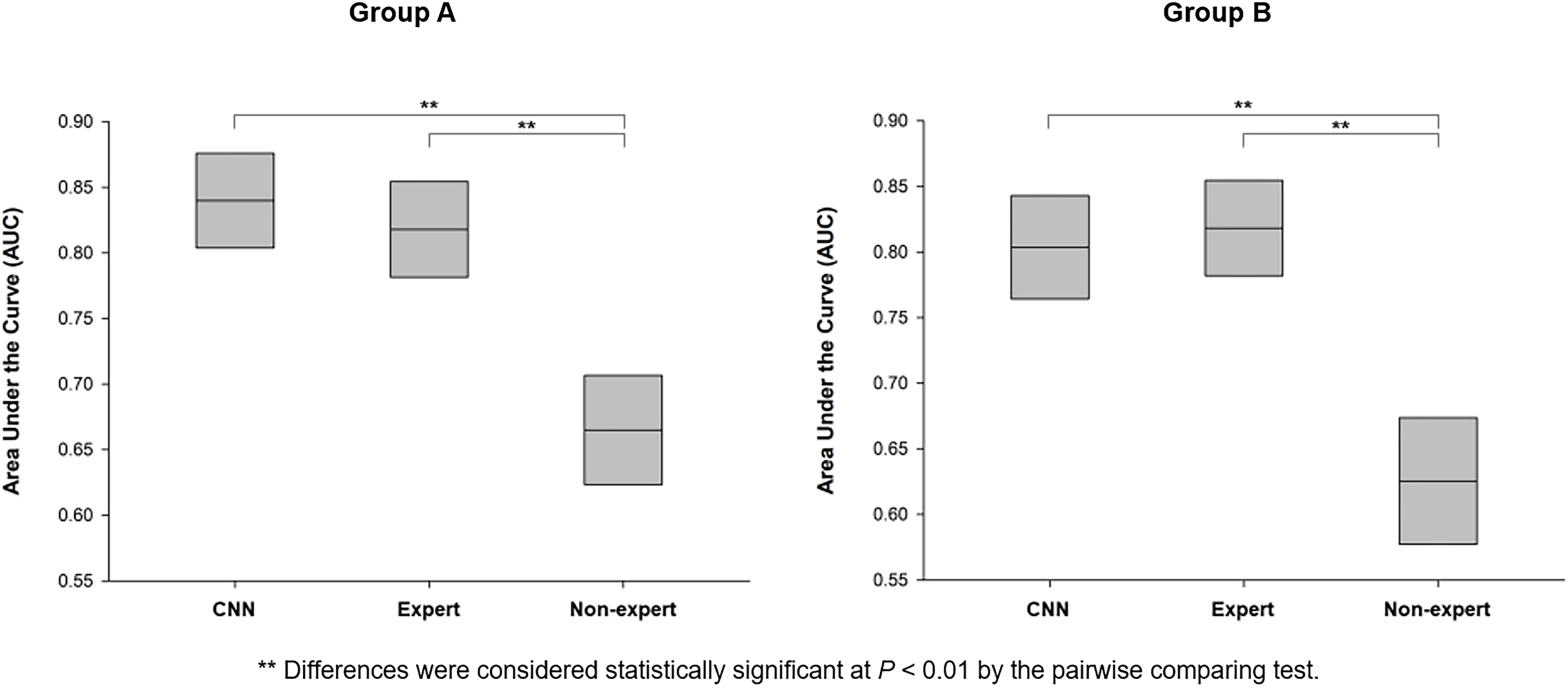
Comparison of diagnostic reliability based on the area under the curve (AUC).

In the group A results obtained by the training of Group B images, CNN showed 92.57% sensitivity and 75.39% specificity, which were similar to those of the expert (94.88% and 68.72%, respectively). However, the non-expert showed lower sensitivity (41.71%) and relatively higher specificity (91.28%, Table 2). For diagnostic accuracy, both the CNN and expert group showed similar scores (83.51% and 81.08%, respectively), which were higher than that of the non-expert (67.84%, Fig 5). In the result of group B by the training of group A images, CNN also showed a higher diagnostic accuracy (80.23%) than that of the non-expert (62.71%) but was similar to that of the expert (81.64%). For validating diagnostic reliability, both the CNN and expert showed an AUC above 0.8 in group A and B (Fig 5). However, the non-expert showed a lower AUC (Group A: 0.66, Group B: 0.63). In the calculation of Youden’s index, CNN showed 0.6795 in group A and 0.6073 in group B, which were similar score with the expert (group A: 0.6358, group B: 0.6365) and higher than non-expert (group A: 0.3899, group B: 0.2509).

**Table 2:** Comparison metrics among CNN, expert, and non-expert.

			Value	95% confidential interval
Group A (n = 362)	Sensitivity (%)	CNN	92.57	87.63–95.96
		Expert	94.88	90.46–97.62
		Non-expert	41.71	34.32–49.39
	Specificity (%)	CNN	75.39	68.72–81.26
		Expert	68.72	61.71–75.15
		Non-expert	91.28	86.41–94.84
	PPV (%)	CNN	77.14	72.46–81.24
		Expert	73.13	68.79–77.07
		Non-expert	81.11	72.52–87.48
	NPV (%)	CNN	91.88	86.95–95.05
		Expert	93.71	88.67–96.59
		Non-expert	63.57	60.45–66.58
	Accuracy (%)	CNN	83.51	79.39–96.94
		Expert	81.08	76.78–84.74
		Non-expert	67.84	62.92–72.40
	Cohen’s kappa	CNN	0.6727	0.5989–0.7474
		Expert	0.6262	0.5504–0.7020
		Non-expert	0.3384	0.2526–0.4242
Group B (n = 362)	Sensitivity (%)	CNN	92.57	87.63–95.99
		Expert	98.29	95.07–99.65
		Non-expert	48.00	40.40–55.67
	Specificity (%)	CNN	68.16	60.79–74.91
		Expert	65.36	57.90–72.31
		Non-expert	77.10	70.24–83.03
	PPV (%)	CNN	73.97	69.55–77.95
		Expert	73.50	69.39–77.25
		Non-expert	67.20	60.05–73.64
	NPV (%)	CNN	90.37	84.64–94.12
		Expert	97.50	92.67–99.18
		Non-expert	60.26	56.30–64.10
	Accuracy (%)	CNN	80.23	75.77–84.04
		Expert	81.64	77.27–85.33
		Non-expert	62.71	57.56–67.59
	Cohen’s kappa	CNN	0.6056	0.5254–0.6858
		Expert	0.6341	0.5583–0.7099
		Non-expert	0.2518	0.1550–0.3486

PPV: Positive Predictive Value, NPV: Negative Predictive Value, CNN: Convolutional Neural Network

Regarding the concordance rate between the CNN and expert group, 73 cases (73/362, 20.17%) in Group A (AM: 14 cases, BN: 59 cases) were discordant. Of these, 41 cases (56.16%) of the CNN and 32 cases (43.84%) of the expert were identical with the pathologic results. However, in the concordant cases between them, 29 cases (29/362, 8.01%) differed from the pathology reports. In Group B, 57 cases (AM: 12 cases, BN: 45 cases) showed discordance between the CNN and expert, and 26 cases (45.61%) of the CNN and 31 cases (54.39%) of the expert were identical with the pathologic results. Among the concordant cases in group B, 39 cases (39/362, 10.77%) differed from the pathology results. Cohen’s kappa between CNN and Expert, CNN and Non-expert, Expert and Non-expert is shown in Table 3.

**Table 3:** Cohen’s kappa between CNN and expert, CNN and non-expert, expert and non-expert.

	CNN and Expert (95% confidential interval)	CNN and Non-expert (95% confidential interval)	Expert and Non-expert (95% confidential interval)
Group A	0.5929 (0.5099–0.6760)	0.2620 (0.1868–0.3373)	0.2496 (0.1808–0.3185)
Group B	0.6513 (0.5692–0.7335)	0.1972 (0.1109–0.2836)	0.1999 (0.1189–0.2811)

To verify the performance of CNN architecture for the discrimination of acral melanoma, we perform the deep learning architecture, Inception-V3, in [13], the state-of-the-art publication for the classification of skin cancer. In [13], a single image was used for learning. Meanwhile, we applied multiple images for learning. Thus, we compared Inception-V3 with a single image and Inception-V3 with multiple images to CNN with multiple images. The results are shown in Table 4.

**Table 4:** Comparison metrics among CNN, Inception-V3 with a single image, and Inception-V3 with multiple images. Inception-V3 (s) corresponds to Inception-V3 with a single image and Inception-V3 (m) corresponds to Inception-V3 with multiple images.

			Value (%)	95% confidential interval (%)
Group A (n = 362)	Sensitivity	CNN	92.57	87.63–95.96
		Inception-V3 (s)	80.57	73.92–86.15
		Inception-V3 (m)	86.29	80.29–91.01
	Specificity	CNN	75.39	68.72–81.26
		Inception-V3 (s)	77.84	71.33–83.47
		Inception-V3 (m)	75.77	69.12–81.62
	PPV	CNN	77.14	72.46–81.24
		Inception-V3 (s)	76.63	71.38–81.17
		Inception-V3 (m)	76.26	71.33–80.58
	NPV	CNN	91.88	86.95–95.05
		Inception-V3 (s)	81.62	76.49–85.84
		Inception-V3 (m)	85.96	80.72–89.95
	Accuracy	CNN	83.51	79.39–96.94
		Inception-V3 (s)	79.13	74.69–82.97
		Inception-V3 (m)	80.76	76.43–84.46
	Kappa	CNN	67.27	59.89–74.74
		Inception-V3 (s)	58.26	49.98–66.54
		Inception-V3 (m)	61.66	53.70–69.62
	AUC	CNN	0.84	
		Inception-V3 (s)	0.79	
		Inception-V3 (m)	0.81	
	Youden’s J	CNN	0.6795	
		Inception-V3 (s)	0.5841	
		Inception-V3 (m)	0.6206	
Group B (n = 362)	Sensitivity	CNN	92.57	87.63–95.99
		Inception-V3 (s)	81.71	75.17–87.14
		Inception-V3 (m)	90.28	84.90–94.23
	Specificity	CNN	68.16	60.79–74.91
		Inception-V3 (s)	88.52	82.99–92.75
		Inception-V3 (m)	79.23	72.63–84.86
	PPV	CNN	73.97	69.55–77.95
		Inception-V3 (s)	87.19	81.90–91.11
		Inception-V3 (m)	80.61	75.72–84.71
	NPV	CNN	90.37	84.64–94.12
		Inception-V3 (s)	83.50	78.65–87.42
		Inception-V3 (m)	89.50	84.36–93.09
	Accuracy	CNN	80.23	75.77–84.04
		Inception-V3 (s)	85.19	81.15–88.50
		Inception-V3 (m)	84.63	80.54–88.01
	Kappa	CNN	60.56	52.54–68.58
		Inception-V3 (s)	70.33	62.97–77.69
		Inception-V3 (m)	69.33	61.93–76.74
	AUC	CNN	0.8	
		Inception-V3 (s)	0.851	
		Inception-V3 (m)	0.848	
	Youden’s J	CNN	0.6073	
		Inception-V3 (s)	0.7024	
		Inception-V3 (m)	0.6952	

PPV: Positive Predictive Value, NPV: Negative Predictive Value, CNN: Convolutional Neural Network

## Discussion and conclusions

Although non-invasive and automated diagnostic techniques have been introduced for the early detection of melanoma, they are still not easy to apply in the acral type[7, 19]. This may be due to the overall low occurrence rate of melanoma in Asians, depending on the ethnic differences, which need a longer time to provide a sufficient dataset to improve diagnostic accuracy.

To overcome the problem of an insufficient dataset, we adopt a 2-fold cross validation method, for the training and test groups. In addition, capturing images at different places for one lesion helps to construct a robust CNN. Similarly, data augmentation generating virtual images using rotation, translation, different angle positioning from one image also helps for a robust CNN. These procedures are necessary to construct an automated diagnosis system from small datasets due to the low occurrence rate of acral melanoma. For the effective screening of melanoma, higher sensitivity is required. Thus, if there is a small compartment corresponding to the melanoma in one image, our system considers it as melanoma. Also, our system recognizes one image as one patient.

From the results, the accuracy of the CNN was above 80%, which was similar in both groups and was close to that of the expert. The CNN and expert also showed AUC values above 0.8, indicating good discrimination. Generally, higher AUC values are considered to demonstrate better discriminatory abilities as follows: excellent discrimination, AUC of ≥0.90; good discrimination, 0.80 ≤ AUC < 0.90; fair discrimination, 0.70 ≤ AUC < 0.80; and poor discrimination, AUC of <0.70[20]. Since the AUC of the non-expert was lower than 0.7, CNN can be a useful tool for the early detection of AM by the physicians who are not familiar with the dermoscopic images. Moreover, additional datasets of AM images can improve the diagnostic accuracy of CNN[21], making it a more reliable tool for the evaluation of the need for skin biopsy for hand and feet pigmentation.

There were several auto-classification methods independent of the size of training data using dermatologists’ checklist, such as the ABCD rule and 7-point scale[22–25]. This method used particular features such as color, shape, size, the boundary of the skin lesion, and statistical features of wavelength, which showed 91.26% of accuracy and 0.937 AUC value[25]. However, these cannot be directly applied to acral melanoma due to the different morphologic features such as ridge or furrow patterns. Although there was a new dermoscopic algorithm reflecting these characteristics for diagnosing acral melanoma: BRAAFF[26], it has not yet been applied to the automated diagnosis. In addition, although there is a state-of-art automated classification method for acral melanoma, these methods cannot be generalized and only work well for a particular pattern of acral melanoma, which is a ridge-and-furrow pattern[27]. Automated diagnosis methods using particular features are able to reflect experts’ perception and the speed of performance is fast. However, it is not easy to catch experts’ perception, although we are trying to reach the goal with significant features. On the other hand, deep learning does not require specific features as inputs. It automatically finds the most correlated features with expert’s perception by learning. Thus the accuracy is higher than feature-based methods. However, a large database is critical for the successful completion of deep learning.

Recently, the melanoma classification performance of CNN using 1,010 dermoscopy images was reported as having an AUC of 0.94 [13], which was higher than noted in our results (0.84, 0.8). Our inferior results may be due to the characteristics of AM; it occurs on the pressure area, thick skin, callus, etc., which can hinder and transform the classic pigmented lesion into an atypical case. Because of this, experts in our experiment also showed an AUC of 0.82. Therefore, if the datasets are analyzed separately considering these anatomic characters, CNN may perform a more precise discrimination. Furthermore, if combined with images from non-invasive devices for melanoma diagnosis, which may overcome the problems presented by a thick skin, the accuracy of CNN can be markedly improved.

Several non-invasive devices such as confocal and photon microscopy are being introduced to provide convenient ways to diagnose melanoma early[28]. However, they require much effort and time for a physician to gain expertise. An automated diagnostic system using a CNN, even with a small dataset, may alleviate the difficulty of learning how to use these newly developed devices.

In conclusion, a half-training and half-trial method were useful for creating a comparatively accurate deep-learning model from a relatively small dataset. Although further data analysis is necessary to improve its accuracy, CNN would be helpful for the early detection of AM, which is usually associated with delayed diagnosis and poor prognosis.

## Supporting information

S1 Table STROBE statement. Checklist of items that should be included in reports of observational studies.
(DOCX)
