# Correction: Acral melanoma detection using a convolutional neural network for dermoscopy images

**DOI:** 10.1371/journal.pone.0196621

**Published:** 2018-04-24

**Authors:** Chanki Yu, Sejung Yang, Wonoh Kim, Jinwoong Jung, Kee-Yang Chung, Sang Wook Lee, Byungho Oh

The affiliation of the sixth author is incorrect. San Wook Lee is not affiliated with #3 but with #1 Department of Media Technology, Graduate School of Media, Sogang University, Seoul, Republic of Korea.

Affiliation 3 for Sejung Yang is incorrect. Affiliation 3 should be: Department of Biomedical Engineering, Yonsei University, Wonju, Gangwon-do, Republic of Korea.

The Data Availability statement for this paper is incorrect. The correct statement is: All relevant data are within the paper and its Supporting Information files or are available from figshare at https://figshare.com/s/a8c22c09f999f60a81bd.
